# Incidence and Factors Predicting Skin Burns at the Site of Indifferent Electrode during Radiofrequency Catheter Ablation of Cardiac Arrhythmias

**DOI:** 10.1155/2016/5265682

**Published:** 2016-04-24

**Authors:** Hussain Ibrahim, Bohuslav Finta, Jubran Rind

**Affiliations:** ^1^Grand Rapids Medical Education Partners, Michigan State University, Grand Rapids, MI 49503, USA; ^2^Spectrum Health Medical Group Cardiovascular Services, Grand Rapids, MI 49503, USA

## Abstract

Radiofrequency catheter ablation (RFA) has become a mainstay for treatment of cardiac arrhythmias. Skin burns at the site of an indifferent electrode patch have been a rare, serious, and likely an underreported complication of RFA. The purpose of this study was to determine the incidence of skin burns in cardiac RFA procedures performed at one institution. Also, we wanted to determine the factors predicting skin burns after cardiac RFA procedures at the indifferent electrode skin pad site.* Methods.* A retrospective case control study was performed to compare the characteristics in patients who developed skin burns in a 2-year period.* Results.* Incidence of significant skin burns after RFA was 0.28% (6/2167). Four of the six patients were female and all were Caucasians. Four controls for every case were age and sex matched. Burn patients had significantly higher BMI, procedure time, and postprocedure pain, relative to control subjects (*p* < 0.05, one-tailed testing). No one in either group had evidence of dispersive pad malattachment.* Conclusions.* Our results indicate that burn patients had higher BMI and longer procedure times compared to control subjects. These findings warrant further larger studies on this topic.

## 1. Introduction

Radiofrequency catheter ablation (RFA) has become a mainstay for treatment of cardiac arrhythmias. It is considered a highly effective treatment modality with a low complication rate [1, 2]. Up to 3% of patients undergoing radiofrequency catheter ablation develop major complications from the procedure. These complications include AV block, cardiac tamponade, coronary artery spasm, thrombosis, pericarditis, vascular injury, thromboembolism, TIA or stroke, pulmonary hypertension, pneumothorax, left atrial-esophageal fistula, and phrenic nerve paralysis [3]. Skin burns at the site of an indifferent electrode patch have been a rare, serious, and likely an underreported complication of RFA [4, 5]. Although different studies have looked at incidence and factors that have led to skin burns at the site of skin pad attachment while performing hepatic tumor ablative procedures, [6, 7] literature related to this complication is scant in cardiac arrhythmia ablative procedures. Incidence of skin burns at the site of the indifferent electrode is currently low but it is likely going to increase in future as higher power settings and multiple ablations are more frequently used for ablation of cardiac arrhythmias [8]. Only case reports and case series have been published in the past. This study aims to determine the incidence and different factors predicting possible skin burns at the site of an indifferent electrode patch while performing these cardiac arrhythmia ablative procedures.

## 2. Methods

A retrospective case control study design was used to examine the characteristics in patients who developed skin burns related to the radiofrequency ablation, compared to those who did not develop this complication during the procedure. All patients ≥18 years of age who underwent cardiac RFA procedures from 4/1/2012 to 3/31/2014 that developed skin burns at the site of the indifferent electrode skin patch were included in the review. Controls, defined as patients >18 years of age who underwent cardiac RFA procedures and did not develop skin burns, were randomly selected from the same time frame and matched on age and sex.

Data collected included patient demographics, patient's past medical history (e.g., hypertension and diabetes), height, weight, and BMI. Procedure details (including diagnosis for ablation, type of sedation used, total procedure time, type of generator, maximum temperature reached, and maximum power in watts) were also obtained. Impedance data were not recorded for this patient population and were not included in our study. Indifferent electrode skin patch characteristics (e.g., type, area of attachment, and evidence of malattachment of skin pad at the end of procedure) were collected, as were data related to the characteristics of the burns (e.g., patient complains of pain or not and burn degree (redness, second-degree skin burns, and third-degree skin burns)). Length of hospital management for the burn was also recorded.

Incidence of skin burns from RFA was determined using a query of all patients who underwent cardiac RFA procedures. There were a total of six patients who developed a burn during the study time frame. For the sample size determination, we assumed an odds ratio of 6.0 as clinically important, with 20% of the controls exposed to the risk factor and with *α* = 0.05 and *β* = 0.20. We planned to be able to detect a statistically significant effect with four control patients for every burn patient, using a one-tailed test. The records from 24 control patients and six burn patients were reviewed for this study.

Data were analyzed using IBM SPSS Statistics v 21.0. (Armonk, NY). Quantitative data were analyzed using the Mann-Whitney *U* test and are shown as the mean ± SD. Nominal data were analyzed using Fisher's exact test and are shown as percentages. Significance was assessed at *p* < 0.05, using one-tailed testing.

## 3. Results

Incidence of the significant skin burns after the RFA ablation procedure was found to be 0.28% (6/2167) during the study period. Two of our six burn subjects were males and all were Caucasians. Eight of the 16 control subjects were males, 22/24 were Caucasian, and their age (63.7 ± 8.6 years, mean ± SD) was similar to that of the burn patients (63.7 ± 8.1 years). No significant difference was present between cases and controls with regard to hypertension, diabetes, skin pad, or type of sedation (Table 1). Postprocedure pain was predictably present in significantly more cases compared to controls.

No patient in either group had evidence of dispersive pad malattachment and none of the patients' hair was removed at the site of attachment. Subjects with burns had significantly greater BMI and total procedure time, relative to the control subjects (Table 2). There were no statistically significant differences between the cases and controls with regard to maximum temperature reached or maximum current in watts.

## 4. Discussion

Radiofrequency ablation of cardiac arrhythmias uses low voltage and high frequency electrical energy. During the management of nonarrhythmic conditions like radiofrequency ablation of hepatic tumors, increased level of radiofrequency energy is frequently used, causing higher incidence of potential complications including skin burns at the site of the indifferent electrode patch [9]. Severe skin burns occur in 0.1%–3% of patients undergoing RF ablation of solid abdominal tumors while mild skin burns occur in up to 33% of such patients [6, 10]. This high rate is postulated to be secondary to high power settings and prolonged procedure times during these ablations [11].

Tissue temperature increases with the passage of electric current, with the greatest increase in temperature being at the site of catheter tip, while the temperature increase is attenuated at the site of indifferent electrode site with the help of dispersive skin patches. Increase of temperature at the catheter tip is the foundation of the therapy with RFA as it causes the local destruction of the tissue. Dispersive skin pads at the site of the indifferent electrode function to disperse the electrical energy exiting the body and thus prevent the occurrence of skin burns by spreading the energy over a larger surface area [3]. In prior studies, it has been predicted that the temperature rises to 45–47°C. At the indifferent electrode site, there is a risk factor for development of skin burns [12].

Malattachment of dispersive pads, presence of hair at the site of pad attachment, and increased amount of subdermal fat have been described as known risk factors for development of skin burns. Fat tissue acts like an insulator and increases the temperature secondary to increase in the resistance [9]. Dysfunction of the skin pad by either malattachment or physical damage concentrates the exiting current's available area, resulting in increased tissue temperature and higher risk for development of skin burns.

Our results showed that there was a low incidence of burns at the indifferent electrode skin pad during RFA ablation procedures for cardiac arrhythmias. Nevertheless, this can be a potentially serious complication, as two of our patients developed third-degree burns requiring increased burn care. All of our six burn patients were Caucasians, indicating that there might be a predisposition to develop the skin burns secondary to skin characteristics. However, 22 of our 24 controls were also Caucasians, thus making this association weaker as Caucasian patients seem to be the predominant ethnic group who underwent the RFA procedures at our institution.

BMI is an important factor which can be helpful in predicting the patients' risk of developing skin burns. It is expected that, with increased body weight, there would be more impedance during the RFA procedure, resulting in an increased incidence of the skin burns at the site of the indifferent electrode. Our results indicated that burn patients had significantly higher BMI relative to our control subjects. We suggest that care should be taken in patients who have increased BMI while performing the RFA procedures.

Hair was not removed at the site of the indifferent electrode in our patients, as this might lead to the malattachment of the dispersive skin pad, increased resistance, and thus increased incidence of skin burns which can be associated with it. There was no evidence of malattachment of the indifferent electrode in either the cases or the controls. Another interesting finding was that all patients who had burns had the indifferent electrode applied to the left flank. Among the controls, 12 out of 24 patients had the indifferent electrode attached in the left flank. One patient from the controls had the placement on the left leg, five had it on the right leg, and six controls had the indifferent electrode placed in the right flank region. In a previous study, risk of development of skin burns was found to be lower in patients with the dispersive skin pad attached to the thigh as compared to other body parts [13]. Optimal position of the dispersive skin pad needs to be studied further as it seems to be an important risk factor that is easily modifiable.

Four out of our six cases with the skin burns complained of the pain at the site of indifferent electrode placement. Further, these patients had developed second- and third-degree skin burns. Pain assessment at the end of the procedure for the patients who are under conscious sedation and at the time of becoming conscious for those who undergo the procedure under general anesthesia can be helpful for actively looking for the skin lesions in a timely manner.

Total procedure time was significantly higher in burn patients relative to control subjects, suggesting that it may be a clinically important factor of predicting skin burns. Increased total procedure time indicates technical difficulty of the procedure, patient characteristics that are unfavorable, and/or a difficult to treat arrhythmia requiring increased duration of the procedure to achieve adequate ablation.

Maximum temperature reached during the procedure did not seem to have a major impact on our study sample. This points towards the fact that sustained ablation for longer period of time is more likely to cause the skin burns than higher temperature for shorter periods of time. Thus, one of the important steps in reduction of the post-RFA ablation skin burns is to not prolong the ablation procedure.

Impedance is an important factor in radiofrequency ablation procedures and its monitoring can be helpful in predicting development of skin burns. It is the weighted average of electrical resistivity of all the tissues between the ablation catheter and the indifferent electrode patch. Regions closer to the radiofrequency ablation catheter have the highest weightage in determination of impedance because of high electrical density [14].

Important determinants of impedance include increased body surface area, blood flow to the tissues, coagulum, and char formation. Volume of resistive medium between the two electrodes is proportional to the impedance. Thus, obesity and larger body surface area result in high impedance as subdermal fat acts as an insulator. Power used in radiofrequency ablation of cardiac arrhythmias is proportional to the voltage and inversely proportional to the system impedance [15]. Thus, if impedance increases, in order to deliver the same amount of energy to the cardiac tissue, higher power settings are needed. As studies have found that high power and prolonged periods of cardiac ablation are associated with higher incidence of skin burns, [9] it can be concluded that the high impedance is a risk factor for development of skin burns and would be interesting factor to look at in future studies.

Steam popping is another important phenomenon. During the generation of resistive heat during radiofrequency ablation of the cardiac arrhythmias, cardiac tissue fluid can vaporize, forming steam bubbles which can potentially burst open with an audible pop with continued ablation (generally occurs at tissue temperatures above 100°C). An important potential complication associated with this phenomenon is cardiac perforation. Steam popping and vaporization have been associated with a drop in the impedance [16, 17]. The frequency of the steam pops has been noted to be high with higher power [18]. Theoretically, steam popping can be used as a predictor of skin burns which also can occur at high power settings. This has not been described in the literature before and can be looked at in future studies as a potential predictor.

Further studies are needed to assess other possible predictors including impedance and voltage and for confirmation of our results. Establishment of these predictors would help decrease this possible complication, which can be a major issue in many patients.

## 5. Limitations

An important limitation of the study is its small sample size. Another important limitation is the fact that we did not have the complete records of the important possible predictor of voltage used during the procedure. Therefore, we could not assess any association with it. We used a case control study design, which could raise concerns as to whether there might be other differences between the cases and controls which could be driving the significant differences seen in this study. Finally, a decision was made at the time of writing the protocol to use one-tailed testing for all of the statistical analyses.

## 6. Conclusion

This is the first comparative study reporting on skin burns following RFA. Although the sample size was small, burn patients had significantly higher BMI, procedure times, and postprocedure pain relative to control subjects. While larger studies are needed to confirm these findings, these results should be kept in mind when planning to perform RFA.

## Figures and Tables

**Table 1:** Comparisons for nominal variables between the cases (burn patients) and the controls^1^.

Characteristic	Cases (%)	Controls (%)	*p* value
Hypertension	5/6 (83.3%)	20/24 (83.3%)	0.746
Diabetes	2/6 (33.3%)	5/24 (20.8%)	0.433
Postprocedure pain	4/6 (67.7%)	0/24 (0%)	0.001
Type of skin patch			0.545
3M stockert skin patch	4/6 (66.7%)	14/24 (58.3%)	
Valley Lab skin patch	2/6 (33.3%)	10/24 (41.7%)	
General anesthesia	4/6 (66.7%)	13/24 (54.2%)	0.469

^1^The one-tailed Fisher's exact test was used for the analyses.

**Table 2:** Comparisons for quantitative variables between the cases (burn patients) and the controls^1^.

Characteristic	Cases (%)	Controls (%)	*p* value
BMI	36.6 (27.7–65.0)	30.6 (17.6–52.6)	0.044
Procedure time (min)	224.5 (63–332)	122.5 (23–357)	0.035
Maximum temperature (°C)	55.0 (50.0–65.0)	50.0 (40.0–70.0)	0.078
Maximum current (watts)	60.0 (35.0–70.0)	50.0 (35.0–100.0)	0.325

BMI: body mass index.

^1^The one-tailed Mann-Whitney *U* test was used for the analyses.
